# Preimplantation Genetic Screening and The Success Rate of *In Vitro* Fertilization: A Three-Years Study on Iranian Population

**DOI:** 10.22074/cellj.2021.6784

**Published:** 2020-04-22

**Authors:** Mehdi Totonchi, Babak Babaabasi, Hadi Najafi, Mojtaba Rezazadeh Valojerdi, Poopak Eftekhari-Yazdi, Lila Karimian, Navid Almadani, Anahita Mohseni Meybodi, Morteza Kimiai, Mehri Mashayekhi, Tahereh Madani, Hamid Gourabi

**Affiliations:** 1. Department of Genetics, Reproductive Biomedicine Research Center, Royan Institute for Reproductive Biomedicine, ACECR, Tehran, Iran; 2. Department of Stem Cells and Developmental Biology, Cell Science Research Center, Royan Institute for Stem Cell Biology and Technology, ACECR, Tehran, Iran; 3. Departments of Genetics, Faculty of Biological Sciences, Tarbiat Modares University, Tehran, Iran; 4. Department of Embryology, Reproductive Biomedicine Research Center, Royan Institute for Reproductive Biomedicine, ACECR, Tehran, Iran; 5. Department of Endocrinology and Female Infertility, Reproductive Biomedicine Research Center, Royan Institute for Reproductive Biomedicine, ACECR, Tehran, Iran

**Keywords:** Array Comparative Genomic Hybridization, Assisted Reproductive Technology, *In Vitro* Fertilization, Preimplantation Genetic Screening

## Abstract

**Objective:**

*In vitro *fertilization (IVF) is one of the most efficient approaches within the context of assisted reproductive technology (ART) to treat infertility. High pregnancy rates have become the major index of successful IVF in clinical studies. It is not clear yet which factors are certainly responsible for IVF success, as various outcomes were obtained in different IVF centers with different settings. In this study, we aimed to address controversies in the interpretation of promising results of IVF with respect to preimplantation genetic screening (PGS).

**Materials and Methods:**

In this retrospective case series study, we built a dataset containing data from 213 IVF patient candidates for PGS (654 embryos) with blastomere biopsy at day 3 and trophectoderm biopsy in day 5, referred to Royan Institute, Tehran, Iran from 2015 to 2018. Next, the data were analyzed to find influential factors affecting success rate of ART cycles.

**Results:**

Data analyses showed that regardless of PGS indications (ART failures, recurrent miscarriage, chromosomal abnormalities, etc.), the pregnancy rate is influenced by maternal and embryonic factors such as the age of mother as well as quantity and quality of transferred embryos. Furthermore, genotyping of embryos using array comparative genomic hybridization (aCGH) depicted the highest rate of chromosomal aberrations for chromosomes 1, 16 and 19 while the lowest frequency for chromosomes 11 and 17. Similarly, we detected 463 genetically abnormal embryos by aCGH, among which only 41.9% could be detected by classical fluorescent in situ hybridization (FISH) method.

**Conclusion:**

This study not only highlighted the advantages of aCGH over the FISH method in detection of chromosomal abnormalities, but also emphasized the importance of genetic abnormality as an indication for determination of IVF success rate.

## Introduction

Higher pregnancy rate following application of assisted reproduction technology (ART) is probably the main aim of almost all *in vitro* fertilization (IVF) centers. Recent surveys have estimated an average success rate of ~30% for ART, around the world (1, 2). One approach toward higher pregnancy rate is to recognize factors that influence IVF procedure, although it is still a matter of debate (3, 4). In addition to the type of IVF settings, genetic background (such as aneuploidy) of the transferred embryos can potentially affect pregnancy success rate (5). In fact, chromosomal abnormalities of embryo, as the form of either numerical or structural, may possibly cause recurrent ART failure, meaning failure of pregnancy from two or three times good quality embryo transfer (6). Therefore, chromosomal abnormalities need to be considered as an important factor which is responsible for the fate of ART-produced embryo (7). These abnormalities can be “inherited” from a parent (such as translocation) or be “de novo” (new to the embryos) (8). Unlike the inherited chromosomal abnormalities, de novo chromosomal abnormalities may occur during IVF procedure (9) and unexpectedly cause failure of ART (10). Therefore, in addition to the procedures of analyzing the parents’ genotype, introducing an optimal procedure for detection of chromosomal abnormalities of transferred embryos is of great importance (11).

Preimplantation genetic screening (PGS) could be regarded as a risk assessment step for identification of numerical and structural chromosomal abnormalities to ensure genomic integrity of the embryo (2). Some studies explained the benefit of fluorescence in situ hybridization (FISH) (12), oligo-arrays, single nucleotide polymorphism (SNP)-arrays (13), quantitative polymerase chain reaction (qPCR) (12) and bacterial artificial chromosome (BAC)- array for PGS (14). Nevertheless, they are not able to make distinct and comprehensive analyses of the human genome (15). Accordingly, array comparative genomic hybridization (aCGH) was introduced as a reliable and accessible diagnostic approach to assess 24-chromosomal abnormalities in humans (16).

We designed a retrospective case series study to investigate the effect of PGS on IVF outcome as well as the influence of environmental and genetic factors responsible for pregnancy success rate.

## Materials and Methods

### Study design

The retrospective case series study was conducted among the patients who referred to the Royan Institute Infertility Clinic (Tehran, Iran) as IVF candidates from 2015 to 2018. Overall, 213 individuals were chosen based on the history of previous ART treatment cycles and genetic background for aCGH analysis. 

Ovarian stimulation and oocyte retrieval were performed by a standard protocol (i.e. long lutealphase pituitary down-regulation). Briefly, the patients were prescribed to start injection of 0.5 mg/day buserelin SC (Superfact, Aventis, Germany) in the luteal phase of menstrual cycle. After confirmation of hypothalamic-pituitary-ovarian (HPO) axis suppression (the serum E2 levels of less than 50 pg/ml, no ovarian cyst on transvaginal ultrasound examination and thin endometrium) buserelin dosage was reduced from 0.5 mg/day to 0.25 mg/day and it was sustained until administration of human chorionic gonadotropin (hCG) (for puncture triggering). The controlled ovarian hyperstimulation (COH) was commenced with administration of recombinant follicle-stimulating hormone (FSH, Gonal F, Serono, Switzerland) or human menopausal gonadotropin (HMG, Menogon, Ferring Pharmaceuticals, Germany) 150 IU/day on the second day of menstrual cycle. Serial ultrasound monitoring and measuring serum E2 levels for evaluation of ovarian response and adjusting gonadotropin dosage is required. With reaching three follicles diameter to 18 mm, 10,000 IU of recombinant hCG (Pregnyl, Organon, Netherlands) was administered. Oocyte retrieval, by the transvaginal ultrasound guided approach, was performed 34-36 hours after hCG injection.

Oocytes were classified according to their capability for being IVF recipient. As soon as reaching the thickness of uterus to 8 mm with three-line pattern, the patient was treated with progesterone and the treatment was terminated in the case of no pregnancy. Otherwise, the patient continued progesterone treatment during gestation according to Gyenocologist recommendation.

Afterward, all patients were followed-up after PGS and embryo transfer. Ethical approval was obtained from Royan institute to use patients’ data (Ethical code: EC/1393/1082). Variables such as age, history of previous ART failure, recurrent miscarriage (RM), biopsy method, total number of transferred embryos, the day of embryo transfer, embryo quality, infertility etiologies and chromosomal abnormalities of parents were analyzed. Total number of transferred embryos included those for which genotype was performed by aCGH and the others which had good post-IVF quality but had clearly ascertained genotype. Possible IVF confounding factors that may influence the IVF efficiency were also assessed and accurately categorized data were collected.

### Karyotyping

Karyotype analysis for parents was performed on trypsin-banded metaphase chromosomes according to the modifications of Verma and Babu (17). The analysis was performed by a standard protocol to generate a resolution of 550 bands per haploid set, from a single cell of the corresponding parents (18). Normally, 30 random metaphase spreads per sample were targeted. In karyotyping, the result was reported based on the latest International System for Human Cytogenetic Nomenclature (ISCN) (19).

### Sperm preparation

Semen collection was performed mostly by masturbation. For the intracytoplasmic sperm injection (ICSI) procedure, spermatozoa were prepared by the standard swim-up assay. In final sperm suspension, 10% Albuminar-5 (containing 5% human serum albumin, Blood Research Center, Iran) was added to Ham’s-F10 culture medium (Sigma-Aldrich, USA). The prepared spermatozoa were incubated at 37˚C and 6% CO_2_ until the usage.

### Oocyte and embryo culture

The oocyte-cumulus masses were collected in a drop of Ham’s F-10 medium, supplemented with 10% Albuminar-5. Then, the cells were washed in the G-1™ver3 (Vitrolife, Sweden) supplemented with 10% recombinant serum albumin (rHA, Vitrolife, Sweden). In the next step, they were transferred into a 20 μl fresh G-1™ver3 medium and kept under mineral oil in the culture dish. The oocytes were then inseminated with 50,000 spermatozoa/ml and incubated at 37˚C, 6% CO_2_ for overnight.

To proceed fertilization, we used ICSI technique. The oocytes were immersed in the HEPES (Sigma-Aldrich, USA) Ham’s F-10 medium, supplemented with 10% Albuminar-5 and washed in the G-1™ver3 supplemented with 10% rHA. Then, they were transferred into a 5 μl fresh G-1™ver3 medium, kept under mineral oil *in vitro*. The embryos were maintained in G-1™ver3 medium for three days and they were transferred into G2 (G-2TMver 3, Vitrolife, Sweden) from day-3 to day-5. On average, 18 hours post-insemination, the occurrence of fertilization was confirmed. Then, the successfully fertilized oocytes were individually kept in 50 µl drops of embryo culture medium (G.1.2, Vitrolife, Sweden) surrounded by paraffin oil (Sigma-Aldrich, USA) for a maximum period of six days. 

### Embryo evaluation

The cleavage-stage was evaluated on day-3, as previously described by Gardner and Balaban (20). The quality of embryo was scored based on Veeck’s published criteria (21).

Additionally, the quality of embryos was determined according to their appearance characteristics such as shape, size, cell number and integrity of zona pellucida (18). At the time of biopsy, on day-3 post-insemination, the embryos were graded from the highest to lowest quality, A to D, respectively. At the time of transfer, on day-5 post-insemination, embryos were categorized in the following groups: "excellent" (blastocyst, expand blastocyst and hatching blastocyst), "good" (for morula, early and mid-blastocyst) and "poor" (for the other stages). Embryos with spurious appearance were categorized as "not determined (N.D.)". 

Clinical pregnancy was confirmed when an intrauterine fetal pole with a positive fetal heartbeat was observed.

### Embryo biopsy

#### Cleavage-stage biopsy

Eight cells embryos with less than 30% fragmentation at 66 ± 2 hours post-ICSI were considered suitable for biopsy. Less than five cells embryos with more than 30% fragmentation were discarded. Zona opening was performed by laser beam (22, 23) and single blastomere was biopsied.

### Trophectoderm biopsy

Zona of day-3 embryos was initially drilled by laser beam. Primary criteria for the embryo selection was the same as described for Cleavage-stage biopsy. The embryo culture continued and those developing to the blastocyst stage were biopsied using laser technology as previously described (22, 24). Only well-defined inner cell mass blastocysts with hatching trophectoderm were biopsied. Three to eight trophectoderm cells were biopsied.

After biopsy, the embryos were washed in 1X phosphate buffered saline (PBS, Gibco, USA) and they were transferred to digestion buffer with minimum PBS for further genetic analysis. The lysis and Whole Genome Amplification step was performed by SurePlex®DNA Amplification System (Illumina, USA).

Array comparative genomic hybridization aCGH enabled us to accurately detect copy number variations in each individual cell removed from blastomeres or trophoblasts. In order to perform this cytogenetic technique, the 24sure Microarray Pack version 3.0 (Illumina, USA) was applied according to the manufacturer’s protocol. The array data was read by InnoScan 900 microarray scanner (INNOPSYS, France). The BlueFuse Multi v3.1 (Illumina, USA) was used to analyze the 24sure experiments. We reported the median log_2_ ratio for each chromosome as the index of aneuploidy was analyzed by BlueFuse Multi software. 

### Statistical analyses

Chi-square test was used for comparison of the study groups. In all statistical analyses, a P<0.05 was considered statistically significant. Graph plotting and data analysis were done using GraphPad software (version 6) and Microsoft Excel (version 2013). Pearson correlation coefficient (R^2^) was performed using GraphPad Prism software (version 6) to analyze correlations between the studied variables.

## Results

### Indications for preimplantation genetic screening

**Fig.1:**
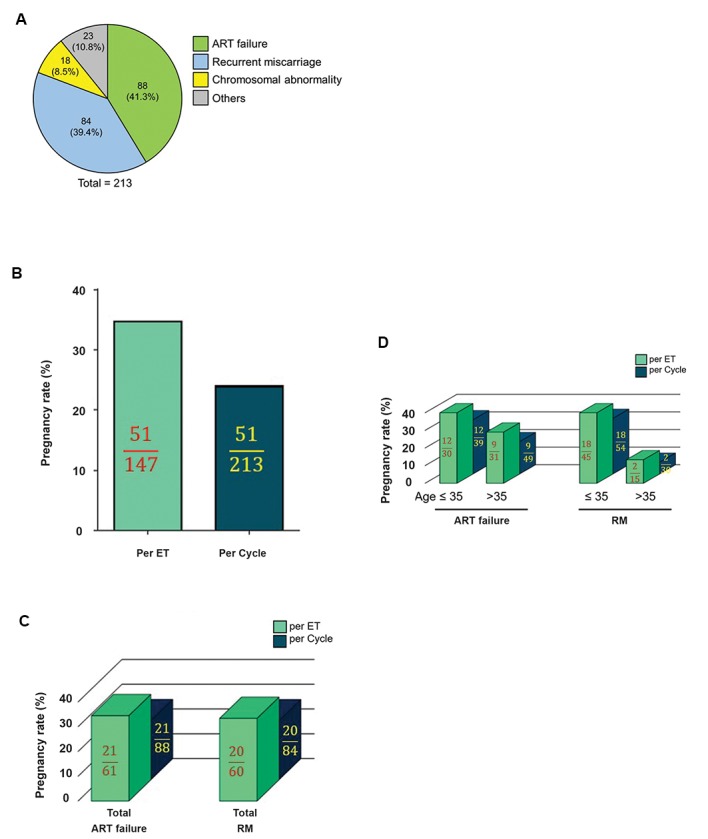
Indications of PGS in IVF patients referred to Royan Institute and its influence on the pregnancy rate. **A.** 213 patients were chosen to undergo PGS by aCGH for several reasons including ART failure (41.3%), recurrent miscarriage (RM, 39.4%) and chromosomal abnormality of parents (8.5%). Some patients who had heterogeneous characteristics (such as advanced age and unexplained infertility) were classified as “others” comprising 10.8% of the population. B. Regardless of the indications of PGS, pregnancy success rate was calculated for IVF and reported as pregnancy rate per ET and cycle. **C.** Pregnancy rate (per ET and cycle) for patients with ART failure and RM. **D.** Evaluating the effect of age on IVF success rate, presented as pregnancy rate per ET and cycle for ART failure and RM groups. PGS; Preimplantation genetic screening, IVF; In vitro fertilization, aCGH; Array comparative genomic hybridization, ART; Assisted reproductive technology, and ET; Embryo transfer. Patients are categorized by their ages: Ages>35 years ≤35.

Indications for PGS such as recurrent miscarriage (RM), ART failure and parental chromosomal aberration, might perhaps justify why PGS was applied following IVF. In this study, patients who had a history of RM, previous ART failures and chromosomal abnormalities were subjected to PGS. Patients with other heterogeneous features (such as mosaicism, advanced maternal age, unexplained infertility, etc.) that were grouped as “others” also underwent PGS. The frequency and percentage of each group are presented as a pie chart. This data shows that most of the patients (80.7%) were subjected to PGS due to ART failure and RM, while patients with chromosomal abnormalities and other features comprised 8.4% and 10% of the total PGS candidates, respectively (Fig.1A).

For a series of 213 cycles, a total of 147 (69%) embryo transfers (ET) were carried out, which resulted in 34.69% and 23.94% of pregnancy rates per ETs and cycles, respectively (Fig.1B). Pregnancy rate between ART failure and RM groups did not show statistically significant difference (Fig.1C).

In addition, clinical pregnancy rates were increased in younger (≤ 35 years old) women (40% versus 29.03% for ART failure, and 40% versus 13.33% for RM groups, Fig.1D, bright bars). Similarly, pregnancies per cycles showed the same pattern in both ART failure and RM groups (Fig.1D, dark bars). Nevertheless, a significant decline was observed in pregnancy rate among the women with >35 years old who also had RM (Fig.1D).

### The effects of embryo transfers number and fresh/ frozen embryos on pregnancy rate

**Fig.2:**
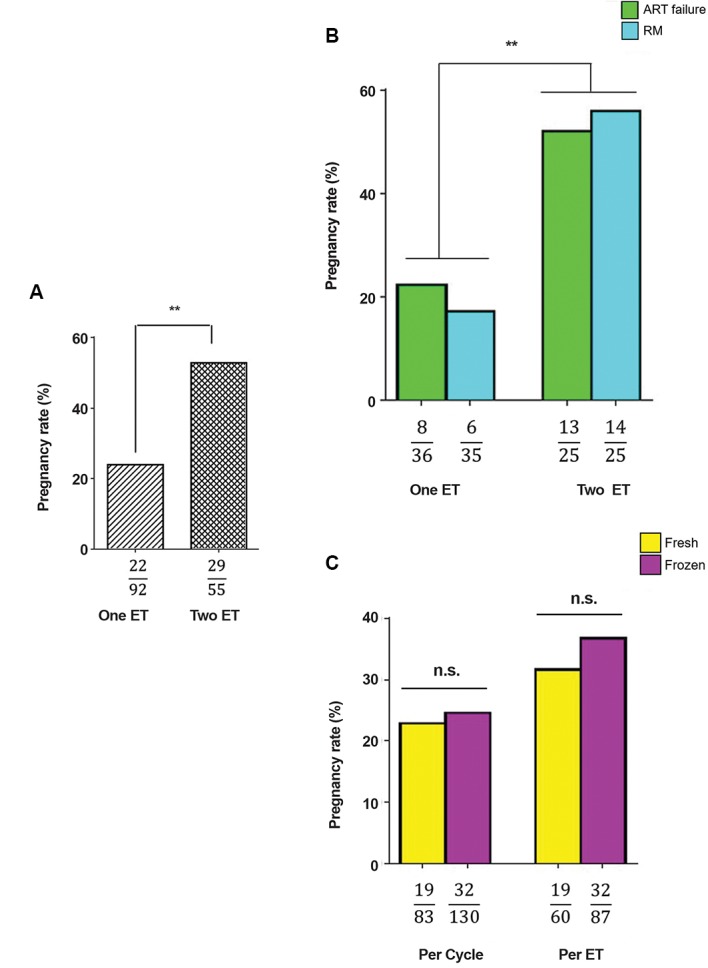
Effect of the number of genetically normal transferred embryos and pregnancy success rate. **A.** Pregnancy rate results were achieved under two conditions: One ET and two ETs. **B.** Pregnancy rate of one ET compared to that of two ETs, separately, for ART failure and RM groups. **C.** Effect of freezing on pregnancy rate was evaluated for the both conditions (one ET and two ETs). Data showed no significant difference in pregnancy rates between fresh and frozen embryos. ET; Embryo transfer, ART; Assisted reproductive technology, and RM; Recurrent miscarriage. n.s; Not significant, *; P<0.05, **; P<0.01, and ***; P<0.001.

Among 213 patients, 147 subjects had at least one healthy embryo (as confirmed by aCGH) undergoing ET. Patients with only one genetically normal transferred embryo (oneET group) had a pregnancy rate of 23.91% (22 out of 92 patients), while patients with two ETs showed a significantly higher level of pregnancy rate (52.72%, 29 out of 55 patients, Fig.2A). This increase in the pregnancy rate for two ETs was also observed in both ART failure and RM groups (Fig.2B).

In order to investigate the influence of fresh or frozen embryos on ART success, the pregnancy rates were calculated for these groups. Results showed the same pregnancy rates per-ET/per-cycle in fresh and frozen embryos (Fig.2C). Figure 2 shows the number of patients (cycles) or ETs, together with positive pregnancy.

### Analyses of chromosomal abnormality rate in embryos undergoing *in vitro* fertilization

**Fig.3:**
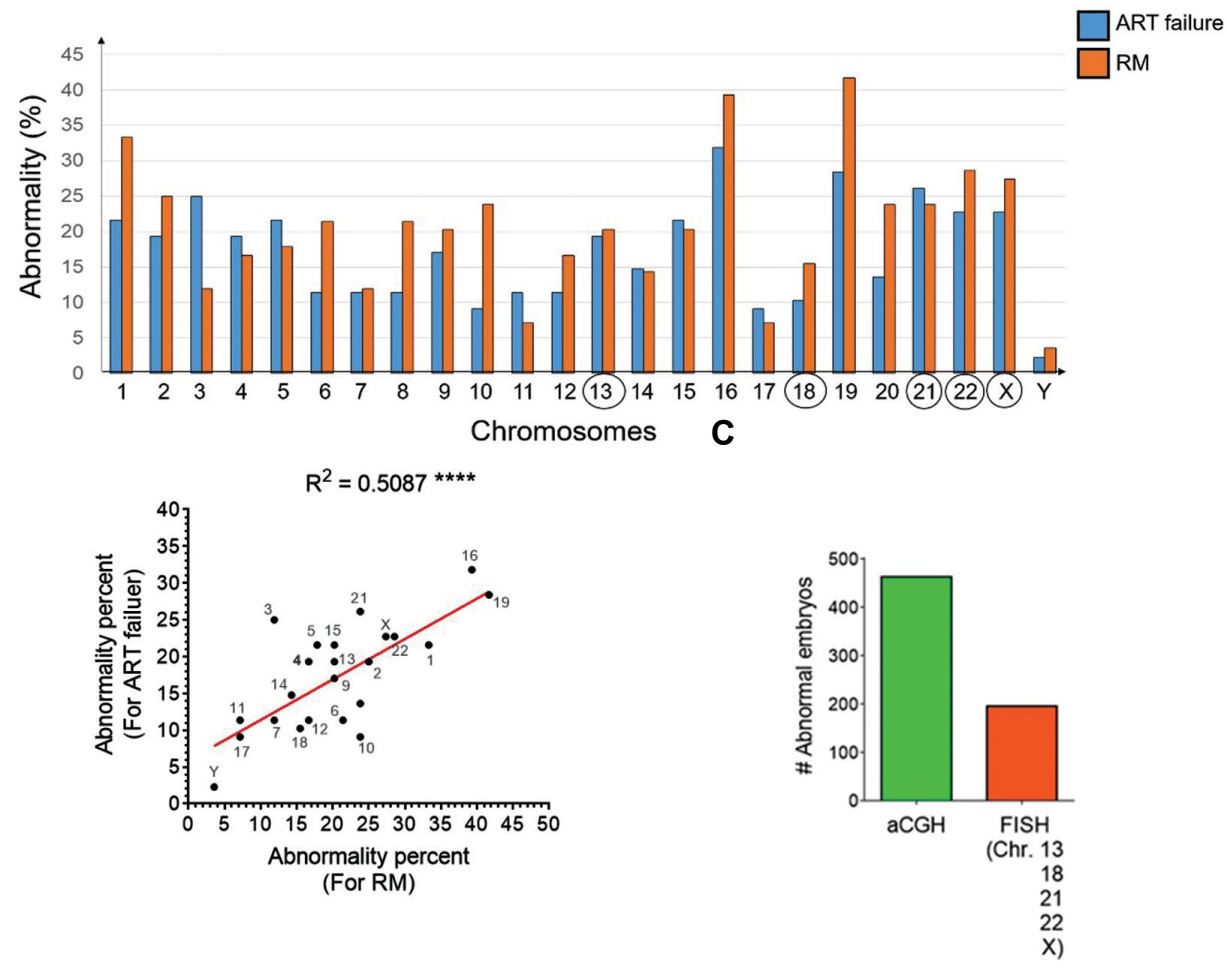
Frequency of chromosomal abnormalities in IVF procedures in addition to efficacies of aCGH and FISH for detection of such abnormalities. **A.** While aCGH surveys the whole genome (24 chromosomes) to detect abnormalities, conventional FISH uses probes that are often specific to chromosomes 13, 18, 21, 22 and X (encircled numbers). Here, aCGH data showed the highest abnormality rate for chromosomes 1, 16 and 19, while the lowest one was found for chromosomes 11 and 17. Chromosomal abnormalities in ART failure and RM groups are presented as blue and orange bars. **B.** Significantly positive correlation between chromosomal abnormality rate in ART failure and RM groups. Each chromosome is shown as a point and abnormality rate of each chromosome for ART failure and RM groups are represented as Y and X axes, respectively. **C.** Comparison of abnormalities, detected by aCGH versus those found by FISH method. IVF; In vitro fertilization, aCGH; Array comparative genomic hybridization, FISH; Fluorescence in situ hybridization, ART; Assisted reproductive technology, and RM; Recurrent miscarriage.

Chromosomal aberration is an inevitable problem during IVF procedure. Therefore, we exploited the advantages of aCGH strategy to explore frequency and type of chromosomal abnormalities occurring during IVF. Analysis of aCGH results showed that the most frequent abnormalities were found in chromosomes 1, 16 and 19 in both ART failure and RM groups. Conversely, chromosomes 17 had the lowest abnormality rates in the two groups (Fig.3A). As it is deduced from Figure 3A, the abnormality rate in both ART failure and RM groups presented a similar pattern. Consistently, a significant positive correlation (R^2^=0.51, P<0.0001) was observed between chromosomal abnormality rates with ART failure (x-axis) and RM (y-axis) groups (Fig.3B). The chromosomes 13, 18, 21, 22 and X (numbers surrounded by circles) are the common ones in PGS using the conventional FISH technique.

In order to compare capability of aCGH and FISH methods to detect different types of such chromosomal abnormalities (i.e. whole chromosome insertion/deletion and partial insertion/deletion), the number of embryos containing chromosomal abnormalities commonly testes by FISH technique (13, 18, 21, 22 and X) were calculated. Our data showed from 463 abnormal embryos detected by aCGH, only 194 embryos could likely be detected by conventional FISH technique (Fig.3C). The rate of partial or complete chromosomal abnormality per abnormal embryos was 2.02 (938 abnormal chromosomes in 463 abnormal embryos).

### The effect of embryo quality and biopsy methods on *in vitro* fertilization succes

**Fig.4:**
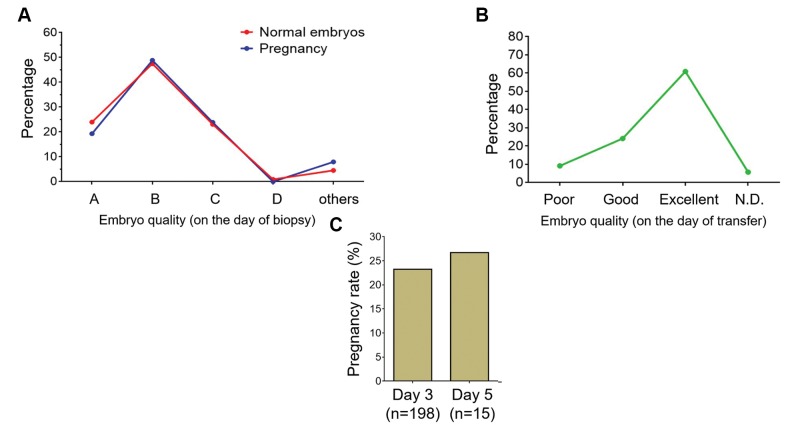
Relationship between the quality of embryos and pregnancy. **A.** Quality of embryos determined on the biopsy days and their relation to the number of normal embryos (red line) and pregnancies (blue line). **B.** Relationship between quality of embryos at the time of transfer and pregnancy frequency (presented in number). **C.** Comparison between the biopsy day (3 or 5) and the rate of pregnancy per embryo transfer (ET).

In addition to the aforementioned factors, other important variables, such as transferred embryos’ quality, may influence the IVF success rate. Thus, all of the tested and transferred embryos were scored based on their quality from “A” to “D” (for the biopsy day) and “poor, good and excellent” (for the transferring day). These scores alongside with IVF assessment results were recorded. The recorded data showed that there is no relationship between the IVF outcome and quality of embryo on the biopsy day (Fig.4A), suggesting that probably grading at the time of biopsy cannot be regarded as a proper measurement to predict IVF outcome. However, assessment at the time of embryo transfer showed a positive relationship between the embryo quality and IVF success (Fig.4B), indicating that embryo grading at this time point is a better determinant for IVF success than scoring on the biopsy day.

In order to investigate influence of the biopsy day (day-3 vs. day-5 of post-insemination) on IVF outcome, pregnancy rate was assessed. The results indicated no significant difference in pregnancy rates between day-3 (23.23%) and day-5 of biopsies (26.67%, Fig.4C).

At both scoring time points, it was difficult to assign a certain quality grade for some embryos with unnatural or unknown phenotypes; therefore, we categorized them as “others” or "not determined" (N.D., Fig.4A, B). However, a pregnancy rate of <10% was observed for these groups. Taken together, these data imply that transferring embryos with higher quality would increase the chance of successful pregnancy

## Discussion

Based on the results of studied cohorts, recognition of the factors that influence the outcome of IVF would benefit clinicians to achieve higher pregnancy rates. Effects of different factors that may influence IVF success rate (mostly referred as clinical pregnancy rate) were investigated in the previous studies (1-4, 25-28). However, since different IVF centers use different approaches, the influence of these factors may vary among different IVF centers. Thus, these variables should be normalized based on various circumstances that exist in different IVF centers.

This retrospective case series study was designed not only to evaluate the factors affecting IVF outcomes, but also to assess the genetic basis of this issue in our IVF center at Royan Institute, Tehran, Iran. Between the years 2015 and 2018, 8650 patients referred to our clinic. Among these patients, only 213 individuals were selected to undergo PGS using the aCGH method, according to the recommendation of the genetic counselor of the center and the final decision of the board of clinicians. Earlier studies concluded that clinical pregnancy rate is generally accepted as the main outcome of IVF (29, 30)

Since PGS candidates had different criteria when referring to PGS laboratory, we were interested in the evaluating relationship of the aforementioned indications with pregnancy rates. Data of this study showed no significant difference between pregnancy rate of the patients with the history of ART failure and RM. Noteworthy, pregnancy rate tended to become lower in aged women, which is consistent with the results reported by previous studies concerning the effect of age on pregnancy rates (25, 31,32). It was also shown while the pregnancy rate is similar among younger women (≤35 years old) in each category, pregnancy rate differs among older women (>35 years old). Importantly, half of the ≥35 years old women with RM had no normal embryo and the rest showed significantly lower pregnancy rate per transfer. This outcome would suggest that PGS-IVF procedure for women >35 years old who possibly have a history of RM is not as efficient as younger patients. Previous studies indicated decreased IVF success rates in older women through observation of predominantly increased aneuploidy in oocytes (4, 33). Since we discarded embryos with aneuploidy, the observed decrease in pregnancy rate for women of > 35 years old, may be due to the presence of other factors such as lower endometrial receptivity (34).

Apart from age, the number of transferred embryos potentially affects the pregnancy rate (25). We found that patients with two transferred embryos, had higher pregnancy rates (2.2 folds), and the increased pregnancy rate were observed in both ART failure and RM groups. These results are in agreement with previous studies which examined the influence of transferred embryo number and pregnancy rate (35).

Aside from the effect of transferred embryo quantity, there was no significant difference in pregnancy rate between fresh and frozen embryos; however, an insignificant slight increase in pregnancy rate was observed following the use of frozen ones. On the other hand, the other studies provided evidence which is not consistent with our findings, as they reported higher pregnancy rate following the utilization of frozen embryos (36). This inconsistency may be due to the adverse effects of ovarian hyperstimulation and its effects on endometrial receptivity (37, 38). Insignificant increases in pregnancy rate observed for frozen embryos in this study, would suggest that IVF outcome for frozen or fresh embryos presumably depends on different factors, such as freezing and thawing procedures and more importantly the condition of patient endometrium. In addition, the PGS candidates were selected based on having a history of ART failure, RM and chromosomal aberration; thus, other factors may simultaneously have an influence on the result of frozen embryo transfer. Therefore, there would be a controversy between our data and previous reports regarding better pregnancy results of frozen embryo transfer (36).

Another determinant for successful IVF is the embryo quality which can be assessed by genotype and phenotype analyses. In this experiment, 654 embryos from all studied patients (213 subjects) were genotyped using aCGH. Results showed that excluding 191 embryos with a chaotic and noisy outcome, 463 embryos had interpretable and meaningful genotyping data. Out of 463 embryos, 195 had abnormalities in chromosomes 13, 18, 21, 22 and X which potentially would be detected by conventional FISH. This data definitely introduces aCGH as a powerful method (rather than FISH) to screen embryos prior transfer process. Our data suggest that quality assessment of the embryos based on both phenotype and genotype can be a good parameter helping us to select a good embryo for IVF. However, there may be some cases in which good morphology of embryos do not harbor favorable genotypes, suggesting necessity of the aCGH method after quality assessment of the embryos (4, 39). In addition, while aCGH cannot detect polyploidies (e.g. triploidy and tetraploidy) and balanced chromosomal rearrangements (e.g. translocations), using complementary tests such as karyotyping and next generation sequencing (NGS) would be useful; nevertheless, they are expensive and time consuming (40).

Data obtained from aCGH also revealed that chromosomes 19, 16 and 1 were frequently aberrant during IVF treatment, while they could not be detected by routinely applied FISH probes for PGS.

Supportive evidence for similar chromosomal abnormality rates in both ART failure and RM groups was also shown by the existence of a significantly positive correlation between chromosomal abnormality rate of ART failure (X axis) and RM (Y axis).

Phenotypic analyses showed that natural appearance of the embryo, at the time of transfer, is an important determinant for successful IVF, whereas determination of embryo status at the time of biopsy does not have such a predictive value. This may be due to the altered quality of embryos during their growth between biopsy and transferring time points.

## Conclusion

Comprehensive chromosomal screening of IVF embryos in parallel with optimizing other factors (such as controlled ovarian stimulation, embryo culture, endometrial receptivity and etc.) not only can increase the pregnancy success rate, but also reduces patient anxiety regarding the abortion, stillbirth and abnormality in offspring.
